# Key elements in the quality assessment of a type 3 medication review

**DOI:** 10.3389/fphar.2023.1258364

**Published:** 2023-10-04

**Authors:** Anneleen Robberechts, Melissa Michielsen, Stephane Steurbaut, Guido R. Y. De Meyer, Hans De Loof

**Affiliations:** ^1^ Meduplace, Royal Pharmacists Association of Antwerp (KAVA), Antwerp, Belgium; ^2^ Laboratory of Physiopharmacology, University of Antwerp, Antwerp, Belgium; ^3^ Research Group Clinical Pharmacology and Clinical Pharmacy, Centre for Pharmaceutical Research, Vrije Universiteit Brussel, Brussels, Belgium; ^4^ Department of Hospital Pharmacy, Jette, Belgium

**Keywords:** medication review, quality assessment, community pharmacy services, pharmaceutical care, community pharmacist, qualitative research

## Abstract

**Background:** Medication reviews are a structured evaluation of a patient’s pharmacotherapy with the aim of optimizing medicines use and improving health outcomes. This entails detecting drug related problems and recommending interventions. A high level of quality is essential for the successful implementation of this service in community pharmacies but currently there is no instrument or tool to assess that overall quality.

**Aim:** This study investigated the development of quality criteria of type 3 medication reviews (MR3s).

**Methods:** After surveying the literature, an electronic questionnaire was developed to gather information about quality criteria for MR3. This survey, in Dutch, was distributed electronically. Four groups were queried: 1) pharmacists, mainly working in the Netherlands, involved in practice research and contacted through the PRISMA (Practice Research In Collaboration With Pharmacists) foundation, 2) Belgian pharmacy academics and pharmacists active in professional associations (APA), 3) Belgian pharmacists trained in medication review (MR) by the Royal Pharmacists Association of Antwerp (KAVA) and 4) Belgian pharmacy students. The survey included 57 criteria, divided into eight domains, which were ranked according to their importance by the participants. The results were analyzed statistically using the nonparametric Kruskal–Wallis test.

**Results:** The survey was completed by 95 participants, including 42 PRISMA pharmacists, 19 APA pharmacists, 18 KAVA pharmacists and 16 pharmacy students. Opinions from participants from the different groups overlapped significantly. The use of simple and understandable language in the conversation with the patient was considered essential by the majority. Discussing the usefulness and purpose of a MR3 with the patient was also rated highly by all groups. Differences of opinion were present in aspects about laboratory values, the use of specific tools, and reporting to and consultation with the treating physician. The participants themselves formulated a limited number of additional assessment criteria.

**Conclusion:** There was widespread agreement on the hierarchy of the quality assessment criteria for MR3s. *Minor* differences were related to the experience of the participants. With these results and a small number of suggested extra criteria, a quality assessment instrument for MR3 can be created.

## Introduction

Following the lead of other countries such as the Netherlands or Australia (Imfeld-Isenegger et al., 2020; Al-Babtain et al., 2022), medication reviews (MRs) are increasingly implemented in primary care in Belgium (Lelubre et al., 2019; Robberechts et al., 2021). As for any new service, quality assessment should be an integral part of their implementation (Moullin et al., 2016; Livet et al., 2019; Rose et al., 2020; Clements et al., 2021; Nesbit et al., 2022). In 2017, the Royal Pharmacists Association of Antwerp (KAVA) started a pilot project in which pharmacists were trained in conducting type 3 MR (MR3) i.e., an advanced or clinical MR. Type 3 MR starts from a complete medication history, adds medical data and includes an extensive interview with the patient as well as feedback from the physician (Griese-Mammen et al., 2018; Robberechts et al., 2021).

In recent years, more focus has been placed on the implementation and quality of MRs (Shrank et al., 2007; Harding and Wilcock, 2010; Krska et al., 2010; Mestres Gonzalvo et al., 2014; Beuscart et al., 2018; Livet et al., 2019; Rose et al., 2020; Clements et al., 2021). Five studies dealt with Medicines Use Review (MUR), a type 2a MR (Beuscart et al., 2018; Rose et al., 2020; McCahon et al., 2021) and two other studies involved type 3 MR (Shrank et al., 2007; Harding and Wilcock, 2010; Livet et al., 2019; Clements et al., 2021) whereas the type of MR was not specified in three other studies (Krska et al., 2010; Mestres Gonzalvo et al., 2014). Only one of the MR3 studies was carried out by pharmacists in primary care (Mestres Gonzalvo et al., 2014). The quality of the MR can be affected by the pharmacist’s competence, guidelines, , willingness to engage in an extended role, organizational setting of the pharmacy (e.g., time available), financial rewards and peer review (Harding and Wilcock, 2010; Mestres Gonzalvo et al., 2014). Rose et al. analysed pharmacists’ activities during MR across six countries (Australia, Canada, Chile, Germany, United Kingdom and United States) (Rose et al., 2020) and found that MRs were not performed in a consistent and standardized way across or within these countries. Activities such as “assess that all medications are optimal” and “follow up with patient”, which are key steps in the patient care process, were not performed in all 6 countries (Rose et al., 2020). Recent research showed that interprofessional collaboration in MR leads to higher quality MRs (Frandsen et al., 2022).

There are various quality improvement initiatives in the wider healthcare setting, such as checklists and tools (Flottorp et al., 2013; Moullin et al., 2016; van Tuijl et al., 2020). These tools aim to facilitate implementation research and quality improvement projects (Flottorp et al., 2013). Additionally, quality measures for pharmacy practice were recently reported to be lacking in terms of development, standardization, and validation (Nesbit et al., 2022). A standardised MR process was also suggested to enable comparisons between process evaluations (Alharthi et al., 2022). Quality parameters may help in the development of a quality assessment tool. However, such a tool should be user-friendly and concise, otherwise it may be ignored. Furthermore, criteria should not be readily predictable, as this can lead to “gaming”, defined as reactive subversion such as “hitting the target and missing the point” or reducing performance where targets do not apply (Bevan and Hood, 2006).

### Aim of the study

This study was undertaken to investigate criteria for quality assessment of MR3s, an aspect that has received little attention in the literature so far. The primary research question focused on identifying the key elements for assessing the quality of a MR3. Furthermore, the study compared the perspectives of various participants on several influencing factors, including experience in conducting MR3s and work setting, to investigate whether specific pharmacist’s characteristics influenced their opinions and to gauge the amount of consensus among the different groups. The objective was to discriminate the relative importance of some topics and inquire if there were any topics we had forgotten.

### Ethics approval

In the Belgian setting, an ethics approval was not required because the survey was anonymous.

## Materials and methods

### Questionnaire design

A comprehensive online survey was prepared after reviewing the literature on the quality assessment of MRs. Literature was consulted until April 2020, when the study was conducted. The questions of the survey were extensively discussed with the researchers M.M., A.R., G.D.M. and H.D.L. All researchers gave feedback on each of the three successive survey drafts. The survey also inquired about the participants’ utilization of specific tools, including the Ghent Older People’s Prescriptions Community Pharmacy Screening (GheOP³s) tool, the START/STOPP criteria, the Medication Appropriateness Index (MAI), and the Opioid Risk Tool (ORT) (Hanlon and Schmader, 2013; O'Mahony et al., 2015; Foubert et al., 2021; Strand et al., 2022). Subsequently, the feedback of three pharmacists who were not involved in the design of the survey was incorporated in the fourth and final version. The Qualtrics online survey tool was used to conduct the questionnaire (Qualtrics, 2020). A translation of the full survey, statements, and original Dutch questions can be found in the appendix.

### Design and content validity of the study

The survey asked participants to rank 57 statements in eight domains for importance, without allowing for ties. Each domain contained five to nine statements that were presented to each participant in an individually randomized order. The survey started with four general questions to determine the profile of the respondent. After finishing the survey, participants were asked if any statements or criteria were missing from the questionnaire and if they had any other feedback.

### Questionnaire distribution

The online questionnaire link was emailed in April 2020 to four different groups: 1) pharmacists involved in practice research and approached through the Dutch PRISMA (Practice Research In Collaboration With Pharmacists) foundation, 2) pharmacy academics and pharmacists active in professional development as well as in insurance companies in Flanders (APA), 3) Flemish pharmacists trained in MR3 by KAVA (Robberechts et al., 2021) and 4) last year pharmacy students with a varied amount of real word experience studying at the University of Antwerp, Belgium. Each group received a separate link to the survey. The PRISMA foundation sent the survey to all of its members and the email to students reached all last year pharmacy students at the University of Antwerp. Targeted communication was used for pharmacists in the APA group, while within the KAVA group all pharmacists who had taken a previous MR3 course were contacted. The survey through PRISMA included an additional question to distinguish the nationalities of the participants.

### Data analysis

Consensus assessment was analysed through the use of bump charts (Kirk, 2016). A statistical evaluation of the results was also performed using the Statistical Package for the Social Sciences (SPSS, version 28.0.1.1, IBM). Differences between groups were evaluated using the nonparametric Kruskall-Wallis test with *p* values < 0.05 pointing toward significant differences of opinion.

## Results

### General results

A total of 113 responses were received. Subsequently, the 18 participants who provided only personal demographic data without responding to statements were excluded. Of these 95 participants, 91 completed the survey in full. Table 1 presents the demographics of the surveyed population that consisted of 59% Belgian, 40% Dutch and 1% German participants. The majority of participants were 31–60 years old, with 34% aged 20%–30% and 8% over 60. The two most prominent participant profiles were pharmacy practice researchers (n = 18, 19%) and pharmacy students (n = 16, 17%). A majority of 78% had pharmacy experience, mainly for 1-5 or 20–30 years.

**TABLE 1:** Demographics of the research population (n = 95).

Measure	Item	Count	Percentage (%)
Age (years)	20–30	32	34
	31–40	19	20
	41–50	18	19
	51–60	18	19
	61–70	8	8
	>70	0	0
Nationality	Belgian (B)	56	59
	Dutch (NL)	38	40
	German (D)	1	1
Group	PRISMA pharmacists (NL + B + D)	42	44
	KAVA pharmacists (B)	18	19
	APA pharmacists (B)	19	20
	Pharmacy students (B)	16	17
Profile	Pharmacy practice researcher	18	19
	Pharmacy student 2nd master	16	17
	Deputy pharmacist (community pharmacy)	12	13
	Practice pharmacist and researcher	9	9
	Head pharmacist (community pharmacy)	8	8
	Academic staff within pharmaceutical care field	8	8
	Researcher in pharmaceutical field	6	6
	Pharmacist involved in professional development of pharmacists	3	3
	Pharmacist stand-in	1	1
	Other	14	15
Years of pharmacy experiences	None	22	23
	1–5	18	19
	5–10	14	15
	10–15	11	12
	15–20	7	7
	20–30	16	17
	>30	7	7
Number of MR3 performed by the participants	None	38	40
	<5	18	19
	5–15	8	8
	16–25	2	2
	>25	29	31

The largest of the four surveyed groups, the PRISMA group encompassing 44% of the participants, displayed considerable heterogeneity, through the inclusion of community pharmacists, researchers and academic teaching staff. Within this group, 39 participants (92%) were Dutch, three (7%) were Belgian, and one participant (2%) held the German nationality. Two groups accounted for respectively 20% and 19% of the participants, namely, the APA group and Belgian pharmacists trained in MR3. The fourth group (n = 16, 17%) consisted of Belgian pharmacy students.

The participants’ experiences with MR3s showed significant variation. Among the participants, 40% had never conducted a MR3, while 31% had completed more than 25 reviews. Notably, the PRISMA group participants exhibited the most extensive experience with MR3. The median time taken to complete the survey was approximately 16 min.

Table 2 displays the statements deemed, on average, to be the most important ones by all participants. No significant differences were found between the groups. For each group, the interquartile range and median for each statement were computed, as detailed in the Appendix.

**TABLE 2:** Overall most important statement within each domain.

Most important statements
- A.1: Pharmacists need to use simple and understandable language with their patients
- B.1: The usefulness and purpose of a MR was discussed with the patient
- C.1: The pharmacist paid attention to whether the patient could swallow the drugs
- D.1: Which drugs was used for which condition was discussed with the patient
- E.1: A recent and clear medication schedule was created and discussed with the patient
- F.1: The pharmacist paid attention to whether all drug indications were still current
- G.1: Patients were given the opportunity to discuss their symptoms with the pharmacist
- H.1: The patient’s expectations and concerns were taken into account when developing the treatment plan

### Ranking of the statements and differences between the groups

The rankings and their variations among the various groups are detailed below and illustrated in Figures 1–9.

**FIGURE 1:**
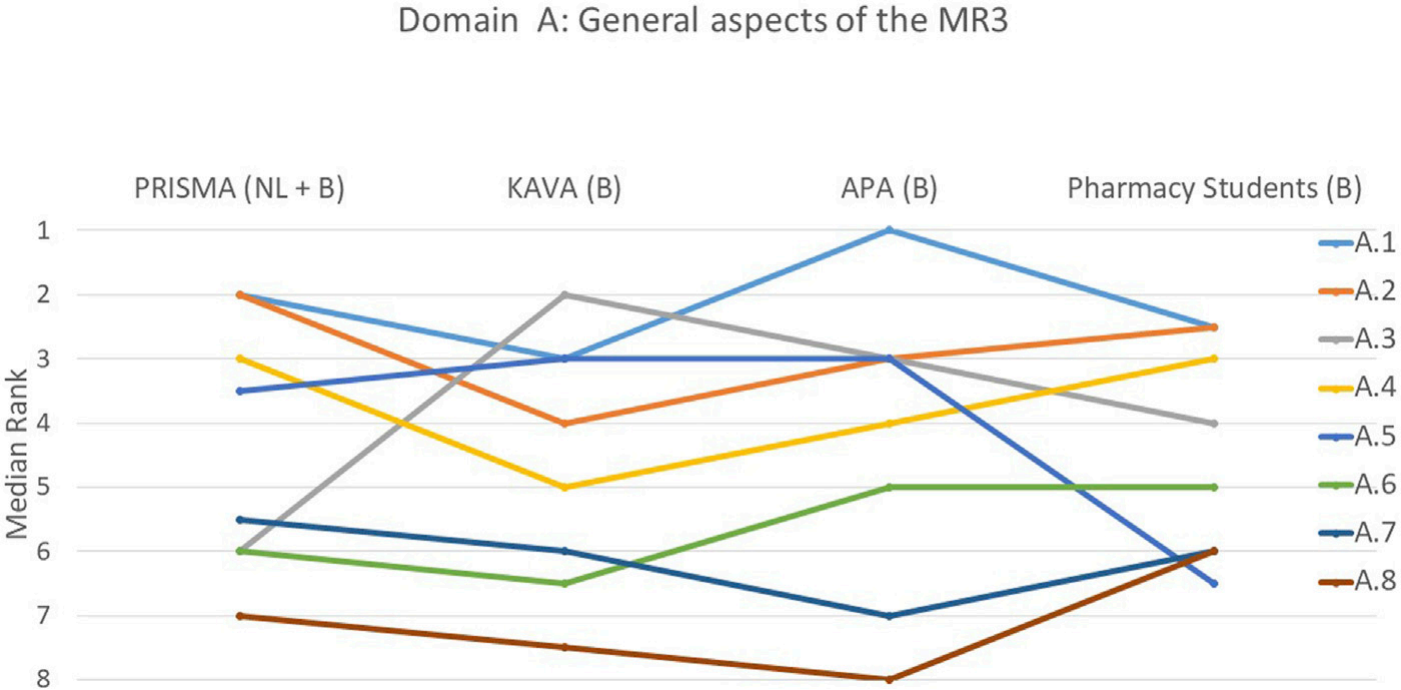
Ranking of the statements from domain A. A.1 Pharmacists need to use simple and understandable language with their patients. A.2 There was reporting and consultation with the attending physician. A.3 Literature consulted and cited was scientific. A.4 Discussion with the patient took place in a familiar and calm environment. A.5 Reliable tools for conducting a MR were used. For example, GheOP³s (Ghent Older People’s Prescriptions Community Pharmacy Screening) (Foubert et al., 2021), START/STOPP criteria (O'Mahony et al., 2015), MAI (Medication Appropriateness Index) (Hanlon and Schmader, 2013), etc. A.6 Consideration was given to communicating non-pharmacological advice. For example, healthy diet, exercise, smoking cessation, etc. A.7 Sufficient attention was given to non-drug substances (dietary supplements, herbal medicines and homeopathy) that the patient may be taking. A.8 Availability of cheaper alternatives for patient and/or National Institute for Health and Disability Insurance (NIHDI) was considered (other drugs, other quantity/packaging).

**FIGURE 2:**
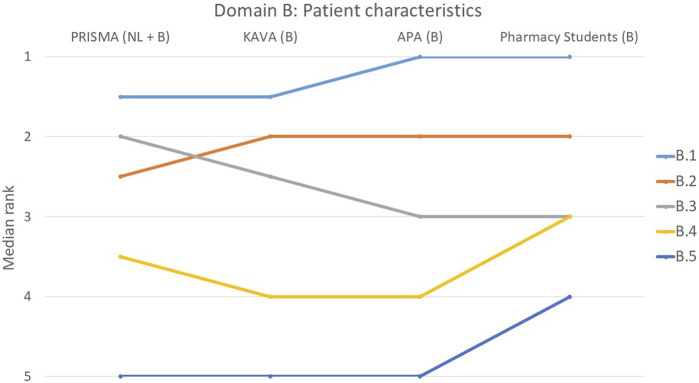
Ranking of the statements from domain B. B.1 The usefulness and purpose of a MR was discussed with the patient. B.2 Patient characteristics at risk for poor adherence were considered. B.3 MR was tailored to the patient’s living situation (informal carer). B.4 Aids used by the patient to perform daily tasks were considered. B.5 Risk of addiction was considered (e.g., by using a screening tool such as ORT (Opioid Risk Tool)).

**FIGURE 3:**
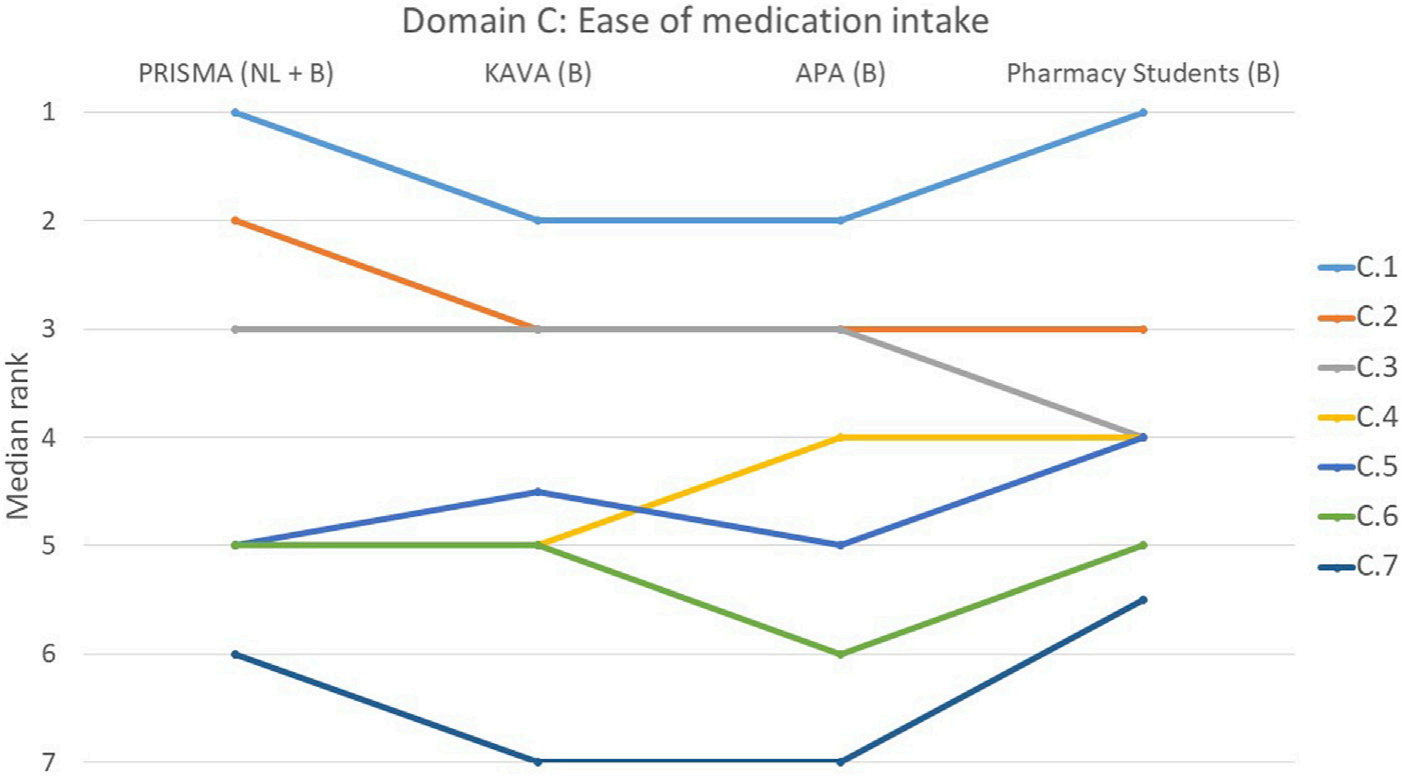
Ranking of the statements from domain C. C.1 The pharmacist paid attention to whether the patient could swallow the drugs. C.2 Pharmacist paid attention to whether the patient can open and close the drugs (including a box, twist-top and/or child-proof closure). C.3 Consideration was given to whether the patient gets his/her drugs out of the blister. C.4 Whether the patient can accurately measure out a liquid was considered. C.5 Pharmacist paid attention to whether the medication could be put into medication boxes. C.6 Proper storage conditions for drugs were discussed with the patient (not in a humid room such as a kitchen, away from children). C.7 Patient’s attitude towards injections was considered.

**FIGURE 4:**
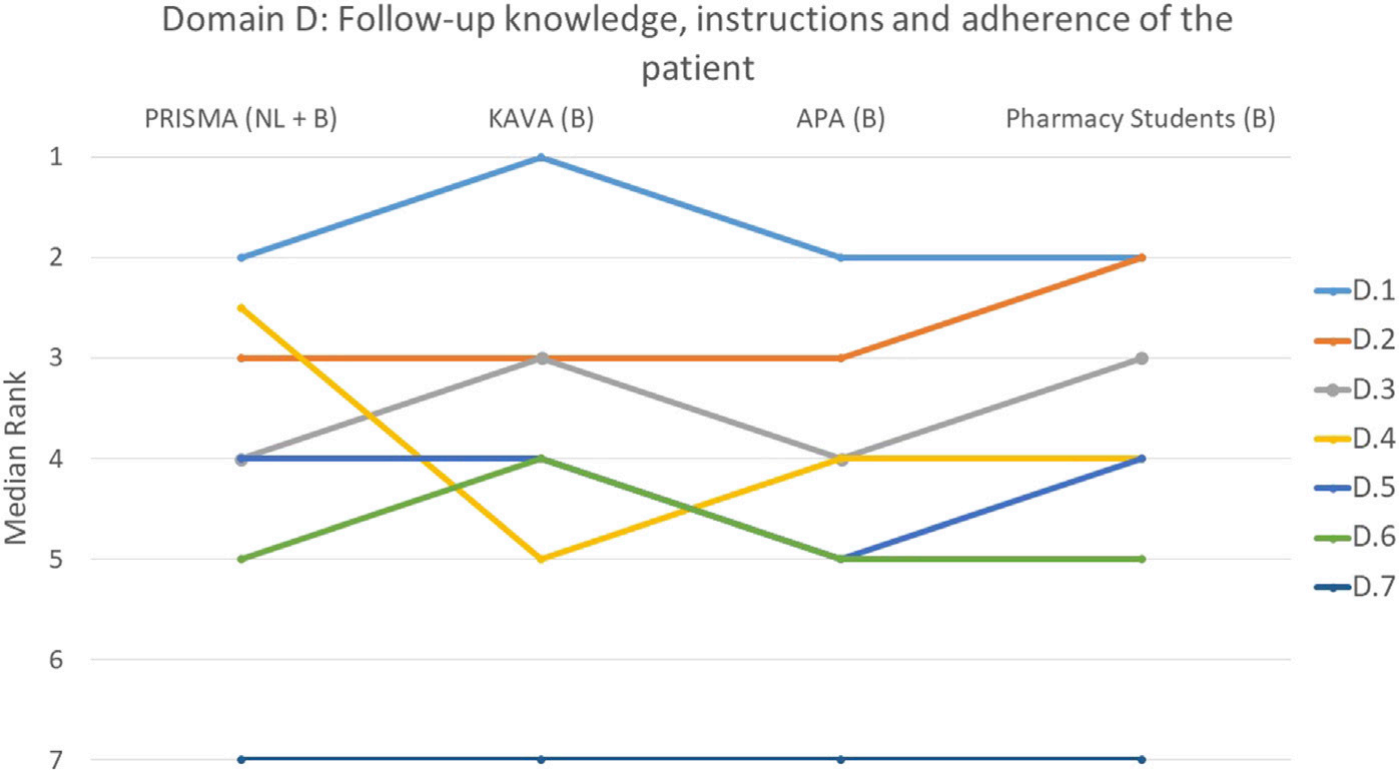
Ranking of the statements from domain D. D.1 Which drugs was used for which condition was discussed with the patient. D.2 Patient was informed about the correct way to take the drug. D.3 Pharmacist paid attention to whether the patient knows the difference between regular drugs and drugs taken only when needed (prn (pro re nata) drugs). D.4 Consideration was given to whether the patient could understand the instructions. D.5 Patient compliance was assessed based on a comprehensive analysis of medication over a sufficient period of time. D.6 Pharmacist paid attention to whether the patient can read instructions. D.7 Pharmacist has paid attention to whether the patient knows when his/her drugs are past their expiry date.

**FIGURE 5:**
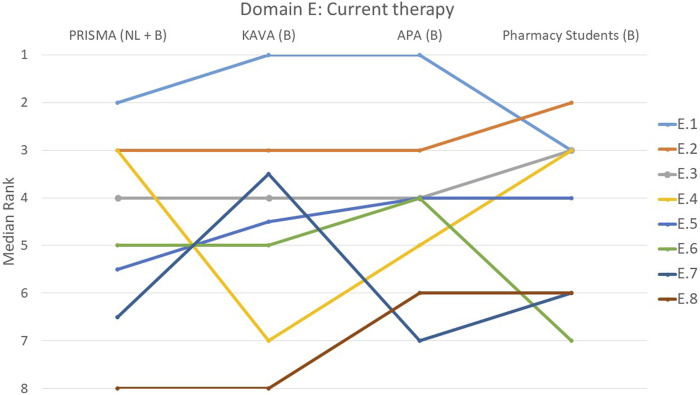
Ranking of the statements from domain E. E.1 A recent and clear medication schedule was created and discussed with the patient. E.2 Consideration was given to which medicines are particularly important for the treatment of the condition. E.3 Pharmacist paid attention to medicines that were missing from the treatment for example, the need for stomach protection, laxatives and/or statins. E.4 Pharmacist paid attention to whether all conditions were treated, if needed for these conditions. E.5 Changes in previous medication use were critically reviewed (why stopped/adjusted?). E.6 Appropriate guidelines were always consulted to evaluate treatment. E.7 Changes to the patient’s previous medication regimen were discussed with them. E.8 Patient's vaccination status was evaluated.

**FIGURE 6:**
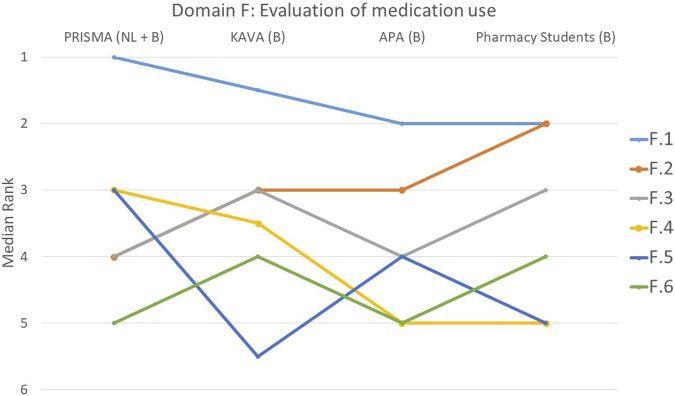
Ranking of the statements from domain F. F.1 Pharmacist paid attention to whether all drug indications were still current. F.2 Drug dose was assessed for appropriateness. F.3 Efforts were made to simplify medication use. F.4 All relevant parameters/lab values requested from the (primary) physician were considered. F.5 A discussion was held with the patient about why certain goals were or were not achieved. F.6 Attention was paid to tapering off the medications.

**FIGURE 7:**
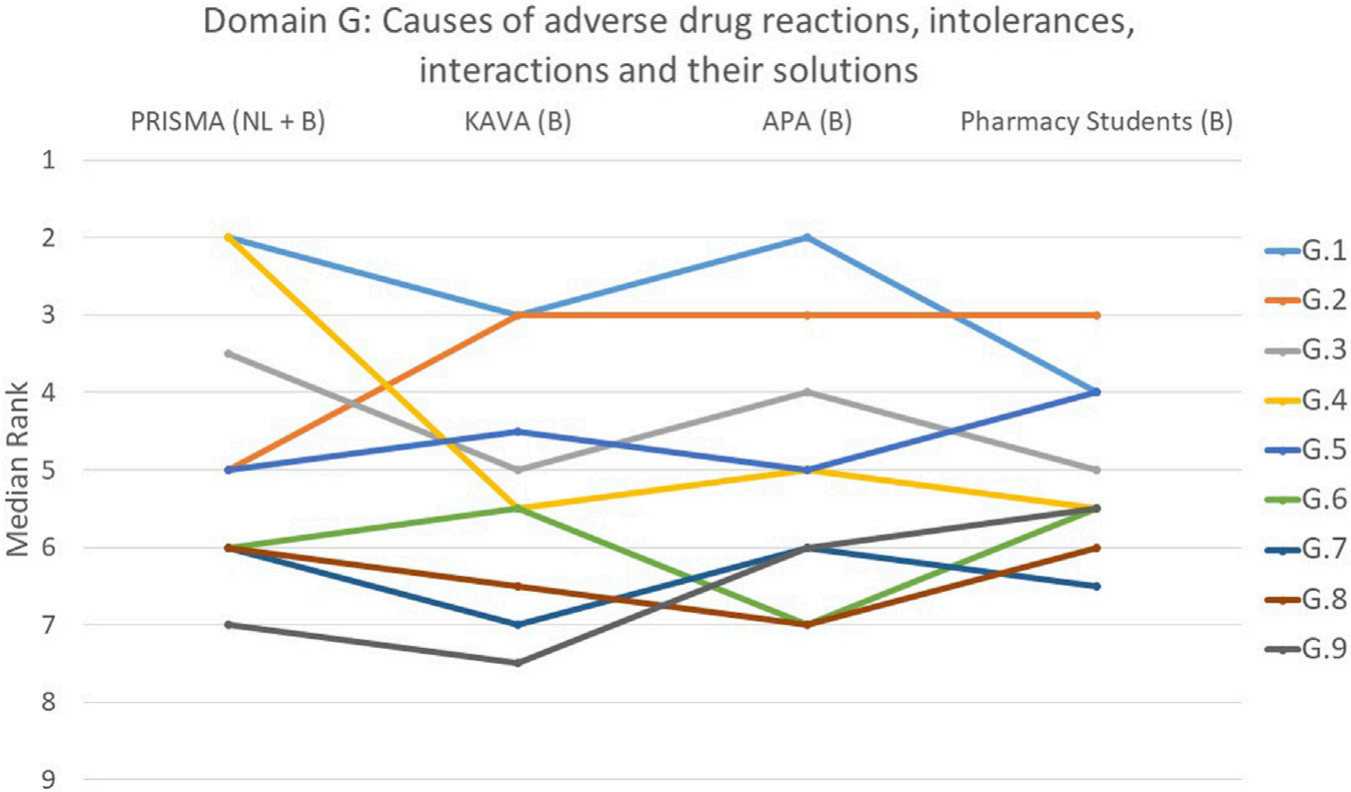
Ranking of the statements from domain G. G.1 Patients were given the opportunity to discuss their illness symptoms with the pharmacist. G.2 The patient was engaged in a discussion regarding the timing of drug intake in relation to nutrition, addressing possible interactions and considerations. G.3 All drugs and their dose were matched to renal function. G.4 During a thorough analysis of the symptoms cited, it was considered whether they could have been caused by the chronic or acute drugs. G.5 Contraindications associated with the condition were identified and evaluated. G.6 Relevant drug-drug interactions between all chronic and temporary drugs were checked and a concrete solution sought whenever necessary. G.7 Pharmacist paid attention to the presence of drugs with central anticholinergic properties. G.8 Patient allergies/intolerances were considered. G.9 Pharmacist paid attention to the presence of QT-prolonging drugs.

**FIGURE 8:**
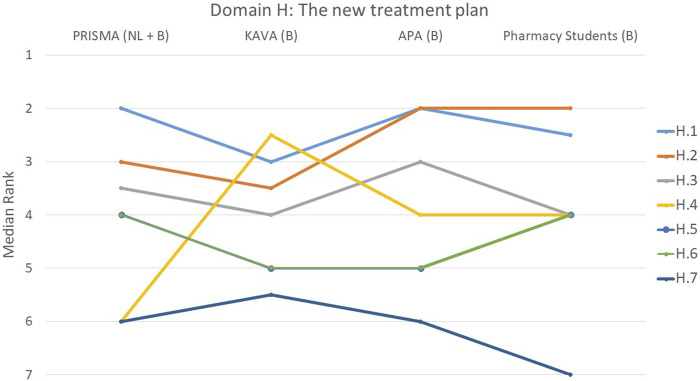
Ranking of the statements from domain H. H.1 The patient’s expectations and concerns were taken into account when developing the treatment plan. H.2 New treatment plan was discussed with the patient and the patient agreed to it. H.3 Clear agreements were made with the patient regarding follow-up. H.4 Patient was informed in detail which over-the-counter (OTC) drugs should no longer be used due to the presence of contraindications. H.5 Clear agreements were made with the physician regarding follow-up. H.6 Priorities in the treatment plan are clear. H.7 Detailed report contains reasoned arguments per recommended adjustment and was reported in writing to the physician.

**FIGURE 9:**
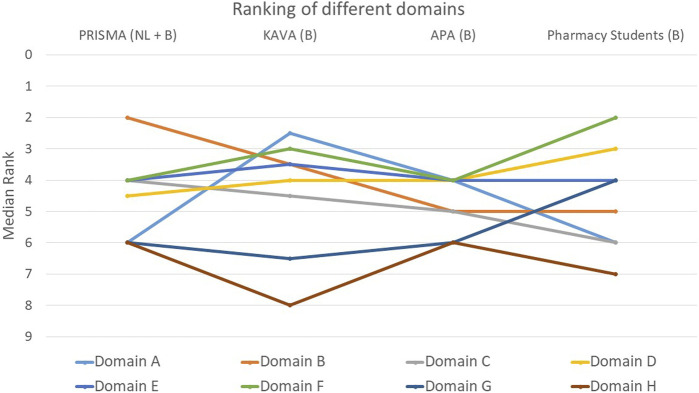
Ranking of the eight different domains. Domain A: General aspects of the MR3. Domain B: Patient characteristics taking into account living situation, resources and need for MR3. Domain C: Patient’s ability: ease of taking, opening drugs and storage conditions. Domain D: The patient: follow-up knowledge, instructions and adherence. Domain E: Current therapy: major drugs, missing drugs, evolution of therapy and vaccination status. Domain F: Evaluation of medication use: simplification, current drug indications, dose appropriateness, use of lab values and achievement of goals. Domain G: Causes of adverse drug reactions, intolerances, interactions and their solutions. Domain H: The new treatment plan: factors for drafting, implementation of adjustments and follow-up.

#### Domain A: general aspects of the MR3

Participants stressed the importance of using language that is easy to understand when communicating with patients (A.1) (Figure 1). There was a significant consensus on this statement, as indicated by a *p*-value of 0.329. Around 58% of participants placed this statement among their top two rankings. Three out of four groups concurred that the statement pertaining to the availability of cheaper medication alternatives for patients and/or the National Institute for Health and Disability Insurance (NIHDI) (A.8) held the least significance with the students’ opinions diverging from this (*p* = 0.007).

However, there was considerable variation in opinions regarding the other statements in this domain, as five of them had a *p*-value below 0.05. A notable difference was observed for statement A.3 that pertained to the use of literature (*p* < 0.001). Participants with less MR3 experience, including KAVA and APA pharmacists and students, deemed it moderately important, while more experienced individuals considered it less important. Another difference between participants with varying levels of MR3 experience was noted for statement A.5 on the use of reliable tools. The student group considered this statement significantly less important (*p* < 0.001), than the other three groups.

#### Domain B: consideration of patient characteristics, including living situation, resources and need for MR3

There was a strong consensus in domain B about the importance of statement B.1 (The usefulness and purpose of a MR3 was discussed with the patient), with 56% of participants ranking it first (*p* = 0.427) (Figure 2). Similarly, statement B.5 that pertained to the consideration of addiction risk, was deemed less important by all groups (*p* = 0.133) and ranked last by 60% of the individual participants. The remaining three statements all had a *p*-value greater than 0.05, suggesting a consensus among the participants.

#### Domain C: the patient’s ability to easily take medication, open drug boxes and store medication under appropriate conditions

In this domain, 55% of participants rated statement C.1 addressing the pharmacist’s consideration of the patient’s ability to swallow medication as most important (*p* = 0.103), while 51% regarded statement C.7 that addressed the patient’s attitude towards injections as the least important (Figure 3). However, students partially dissented about this last statement and ranked it significantly higher than the other three groups (*p* = 0.007). Regarding four other statements (C.2, C.3, C.5 and C.6), there was a consensus among the groups. These statements covered topics such as the ease of opening medication boxes, measuring out liquids, the use of medication boxes and the proper storage of patients’ medicines.

#### Domain D: the patient: follow-up, knowledge, instructions and adherence to therapy

When examining the aspects of follow-up, knowledge, instructions and adherence to therapy, statement D.1 that focused on discussing with the patient which drug was used for which condition, was ranked at the top by 44% of the participants (*p* = 0.222) (Figure 4). Informing the patient about the correct way to take the drug (D.2) was also deemed important by the participants, with 45% ranking it as the most or second most important statement (*p* = 0.210). All groups unanimously agreed that statement D.7, on whether the pharmacist ensured that the patient was aware of the expiration dates of their medications, was the least important (*p* = 0.707). This was indicated by 76% of the participants. A borderline significant difference between groups was only observed for statement D.4 (*p* = 0.048) that focused on the pharmacist’s assessment of the patient’s ability to understand instructions where PRISMA pharmacists, on average, ranked this slightly higher. Despite the presence of divergences, these were not substantial, as illustrated in the figure.

#### Domain E: current therapy: major drugs, missing drugs, evolution of therapy and vaccination status

The creation of an up-to-date and easily understandable medication schedule that was discussed with the patient (E.1), was deemed important by all four groups (*p* = 0.075), with 54% of the participants ranking it as the most important statement within this domain (Figure 5). Statement E.8, on the evaluation of the patient’s vaccination status, was considered the least important by 61% of the participants. However, students once again had a different opinion and considered this statement more important than the other three groups (*p* < 0.001). There was also significant variation on whether the pharmacist paid attention to treating all conditions whenever necessary (E.4) with KAVA pharmacists perceiving this statement as relatively less important (*p* < 0.001). Conversely, for statement E.7 that involved discussing changes to the patient’s previous medication regimen with the patient, KAVA pharmacists rated this as more important than the other groups (*p* < 0.001).

#### Domain F: assessment of medication use, including simplification, verification of current drug indications, evaluation of dose appropriateness, consideration of lab values and determination of goal achievement

With consensus among the groups (*p* = 0.835), 49% of participants considered statement F.1 that focused on the pharmacist’s attention to the current relevance of all drug indications, to be the most important (Figure 6). Statement F.6 that focused on the gradual reduction of medications, was ranked lower by all groups (*p* = 0.123). Statistically significant differences were observed among the four groups for three other statements. The student group ranked statement F.2 that pertained to the drug dosing appropriateness, higher compared to the other groups. In contrast, PRISMA pharmacists indicated that they considered this statement least important during a MR3 (*p* < 0.001). Statement F.4 that involved considering all relevant parameters or lab values obtained from the (primary) physician, was regarded as less important by the students compared to the other groups (*p* = 0.004). Furthermore, the discussion with the patient about why certain goals were or were not achieved (F.5) was deemed much more important by PRISMA pharmacists (*p* = 0.032).

#### Domain G: causes of adverse drug reactions, intolerances, interactions and their solutions

In domain G, the opportunity for patients to discuss their symptoms (G.1) with the pharmacist was ranked as top priority by 39% of participants (*p* = 0.530) (Figure 7). On the other hand, the statement on whether the pharmacist handled QT-prolonging drugs was deemed the least important in domain G (G.9, *p* = 0.150). One-third of participants ranked this statement last or second-to-last. Significant differences between the groups were also observed in statement G.2 that involved the intake of drugs in relation to meals (*p* < 0.001), and statement G.4 that addressed whether the symptoms mentioned by the patients could have been caused by the chronic or temporary use of drugs (*p* = 0.002). PRISMA pharmacists considered G.2 to be less important, while ranking G.4 as more important compared to the other groups.

#### Domain H: the new treatment plan: factors for drafting, implementation of adjustments and follow-up

The statement regarding whether patient expectations/concerns were considered during the development of the treatment plan (H.1) was deemed most important by 31% of participants (Figure 8). Notably, PRISMA pharmacists and pharmacy students demonstrated a tendency to perceive this statement as more important in comparison to the other two groups, although this difference did not reach statistical significance (*p* = 0.388). On the other hand, the least important statement in this domain was H.7 (A detailed report contains reasoned arguments per recommended adjustment and was reported in writing to the physician), with 29% of participants ranking it at the lowest position (*p* = 0.287). This opinion was mainly expressed by the student group with 57% of them ranking this statement last. Regarding statement H.4, on informing the patient in detail about which over-the-counter (OTC) drugs should no longer be used, there was a striking difference between the groups (*p* < 0.001). PRISMA pharmacists considered it the least relevant topic, while KAVA pharmacists scored it a top priority. The other two groups ranked it somewhere in the middle.

#### Global ranking: which of the domains covered do you think is most essential for evaluating a MR3?

When assessing the relative importance of the different domains, participants predominantly selected domain F (follow-up knowledge, instructions and adherence) as the most important (*p* = 0.466): 42% ranked this domain within the top two (Figure 9). In contrast, domains G (causes of adverse drug reactions, intolerances and their solutions) and H (the new treatment plan) were considered the least important by participants. Around 54% of participants ranked domain H last or second-to-last (*p* = 0.011), while for domain G this was 43% (*p* = 0.148). In addition to domain H, domain A (general aspects of the medication review) also exhibited a statistically significant difference between the groups (*p* = 0.011). KAVA pharmacists considered domain A to be much more important compared to the other participants. The remaining three domains, namely, C (ease of medication intake), D (follow-up knowledge, instructions and adherence), and E (current therapy), received an average score in the rankings.

#### Feedback

A number of participants (11%) raised concerns about the ranking method employed, expressing a desire for statements to be grouped together and given equal importance in certain instances.

“The survey was very comprehensive but it was challenging to rank the items because everything appears to be significant in a medication review.”

“My overall feedback on this survey is that it is quite complicated (and seemingly unnuanced) in its design. I’m not sure how useful it is to make a qualitative distinction between many of these statements. I also feel that the format pushed me in a certain direction, so I could not answer honestly or clearly.”

Additional feedback highlighted the components that should be included during each drug dispensing and medication review (MR3). In this context, participants mentioned an ideal scenario where aspects such as adherence and challenges with swallowing are assessed during each pharmacy visit, and a review is conducted to determine if there is still a valid indication for each medication.

“I think certain basic matters such as drug-drug interactions should already have been checked when a medicine is newly dispensed. That's why I ranked these types of checks relatively lower. However, that does not mean they are not important.”

A recurring theme in the comments was the importance of engaging in a conversation with the patient and valuing their input. Four participants emphasized that this dialogue and the patient’s expressed goals should serve as the foundation for the quality of a MR3.

“The pharmacotherapy anamnesis with the patient serves as the cornerstone of every MR, as well as for subsequent analysis and treatment planning.”

“In a MR, it is important to prioritize the patient’s personal goals as much as possible.”

Additionally, there were inquiries regarding time allocation and remuneration.

“Time (and remuneration for it) is not mentioned anywhere, but if anything, it is one of the most decisive factors for doing a MR or not.”

Finally, a participant from the APA group highlighted the significance of the pharmacist’s overall knowledge when conducting a MR3.

“I believe that a good MR is built on the foundation of a pharmacist’s sound basic knowledge. Tools can provide support, but they have little value if the pharmacist lacks understanding what it is all about. In a conversation with physicians, such a lack of knowledge by pharmacists is readily uncovered.”

#### Missing statements

Participants highlighted several crucial factors related to MR3 that were missing. Primarily, there were lingering uncertainties about the appropriate method of patient selection, specifically on how to select patients for whom MR3 is most pertinent. Furthermore, there was a comment to identify patients’ needs concerning MR3, as well as a request to also address aspects of evaluation and monitoring of MR3s, how changes are managed and who is responsible for the evaluation.

“I am missing the comprehensive assessment of the patient’s needs to be the subject of a MR.”

“Evaluation and monitoring, how are the changes going? Who is evaluating this? Does anything need to be adjusted in the treatment plan? Evaluate drugs that may not be effective for certain symptoms? Etc”

## Discussion

### Main findings

The objective of this work was to provide a basis for the key elements of a quality assessment of a MR3. Despite the challenges faced by some participants in ranking the statements (see Feedback and missing statements from the participants), there was a remarkable consensus among the various participant groups in general. This consensus enabled us to identify a statement for each domain that was either partially or fully ranked as the most important across all groups. In 7 out of the 8 (A.1-H.1) statements considered to be of utmost importance, no significant differences were observed between the groups. However, there was one notable exception with divergent opinions about statement C.1 that addressed whether the pharmacist paid attention to whether the patient swallowed the drugs. Even for the statements with the lowest scores, there were substantial similarities between the groups, as 5 out of the 8 statements (A.8, B.5, C.7, D.7, E.8, F.6, G.9 and H.7) did not show any statistically significant difference. Notably, significant differences emerged among the groups for three specific statements: whether patients were asked about the availability of a cheaper alternative (A.8), whether their attitude toward injections was taken into account (C.7), and whether the patient’s vaccination status was evaluated (E.8).

Participants gave high ratings to statements that emphasized the significance of ensuring that patients can easily understand medication reviews. This involved using straightforward and easy-to-understand language when talking to patients (A.1), as well as giving clear, personalized explanations about the benefits and goals of MR3s (B.1). Past investigations have revealed that patients are not always properly informed about the goals or procedures of MR3 (Robberechts et al., 2023). This situation is acknowledged by most respondents, endorsing the approach used to determine crucial elements that demand attention in the continued implementation and quality monitoring of this pharmaceutical care service.

### Differences in opinion between groups

Some divergences were detected in the opinions expressed by the different groups of participants, specifically, PRISMA pharmacists and students demonstrated contrasting rankings on six statements, while KAVA pharmacists showed differences on three statements. However, no significant outliers were identified among APA pharmacists.

### Students *versus* other groups

The importance of using tools was given less significance by the students compared to the other groups (A.5). It is possible that this group either lacks sufficient experience with these tools or, conversely, believes that they possess enough knowledge, making their use redundant. However, none of the groups made the use of these tools a priority as they do not guarantee quality and can lead to differing outcomes, be outdated, or be used incorrectly (Perpétuo et al., 2021). When it comes to ranking, the students gave significantly lower ratings to the report and communication with the physician compared to the other groups (statement H.7). It should be noted that the average student has limited practical experience with MR3s, which may hinder their understanding of the entire process.

The students also assigned a lower ranking to statement (F.4) regarding laboratory values and other parameters provided by the primary physician. The greater availability of such data in the Netherlands (Koster et al., 2016) compared to Belgium easily explains the higher ranking given by Dutch pharmacists.

### PRISMA pharmacists *versus* other groups

The importance of discussing the timing of drug intake in relation to nutrition, including drug-food interactions (G.2), was ranked lower by the PRISMA pharmacists. Feedback revealed that Dutch pharmacists may consider this to be part of regular dispensing rather than something specific to MR3s. PRISMA pharmacists, in contrast to all their Belgian counterparts, gave less weight to the importance of OTC drug use (H.4). PRISMA pharmacists have less data available on the use of OTC drugs by their patients, as these are also available outside pharmacies in the Netherlands (Mertens, 2022) in contrast to Belgium, and where there is a centralized database that includes OTC drugs (FarmaFlux, 2023).

In addition, the PRISMA group also prioritized addressing their patients’ needs more than other groups. This focus on patient-centred care was reflected in their higher rankings for statements such as assessing the patient’s understanding of instructions (D.4), and emphasis on the importance of personal goals (F.5), quality of life, and addressing individualised health problems in the medication management process. (Verdoorn et al., 2019a; Verdoorn et al., 2019b). Finally, PRISMA pharmacists gave a higher ranking to statement G.4 that involved performing a thorough analysis of the patient’s reported symptoms and assessing the likelihood that they were caused by medication use.

### Feedback and missing statements from the participants

Several participants expressed difficulties with the ranking form, citing an inability to assign different statements with the same level of importance. While we understand and empathize with these emotions and worries, in our quest for an overarching ranking, these individual divisions hold no substantial importance. There are antecedents in the literature of the methodological advantages of enforcing a strict ranking without allowing for ties (Krosnick and Alwin, 1988; Blasius, 2012; Moors et al., 2016).

Moreover the substance of specific statements also met with some opposition. Four participants believed that certain statements, for example, about drug-drug interactions, should be implemented and assessed during each pharmacy visit, rather than being discussed specifically within the context of a MR3. Nonetheless, these statements were included in the questionnaire, as there may be situations where specific aspects are overlooked for a range of reasons such as the occurrence of multiple prescribers, frequent hospital visits or dispensing in multiple pharmacies. Overall, the ranking may therefore reflect the participants’ opinion on the benefits of additional monitoring of the patient’s pharmacotherapy during the MR3 process.

Some additional criteria were provided by the participants. One suggestion involved adding a statement about follow-up interviews with the patients. Although this concept was partially covered by statements H.5 and H.6, the explicit mention of conducting follow-up interviews and follow-up adjustments of the treatment plan was absent. Another participant highlighted the importance of relevant patient selection, emphasizing the need for pharmacists to assess and document whether a patient truly requires a MR3 before initiating the process.

Furthermore, participants raised important points regarding the necessity of comprehensive knowledge about pharmacotherapy, the attentiveness to the personal goals of the patients, and the patient’s readiness to participate in the MR3. These factors were also deemed crucial in ensuring the quality of MR3s.

### Similarities and differences in relationship to other studies

There are a small number of studies within the current literature that focus on the quality of MR (Shrank et al., 2007; Harding and Wilcock, 2010; Krska et al., 2010; Mestres Gonzalvo et al., 2014; Beuscart et al., 2018; Livet et al., 2019; Rose et al., 2020; Clements et al., 2021; McCahon et al., 2021; Schröder et al., 2023). We observed a recent increase that coincided with the timing of our survey administration. However, our literature analysis identified divergent interpretations and definitions of MR, making it challenging to compare and synthesize findings across studies, as previously observed by others (Alharthi et al., 2022; Nesbit et al., 2022).

Only one study focused on the implementation of MRs by pharmacists in primary care (Mestres Gonzalvo et al., 2014). The objective of this specific study was to determine pertinent covariates, conducted by a research group comprising 49 participants with expertise in MR. These covariates were rated on a 10-point scale. Our study, which reviewed 57 statements, expanded on Mestres Gonzalvo’s methods and participant selection, providing a broader and more detailed perspective on MR components. This comprehensive analysis provided an even broader and more detailed perspective on the various components involved in a MR.

### Strengths and limitations of the study

One strength of this study is that despite the diverse backgrounds of the participants, there was a high level of agreement on what are the key elements that define the quality of a MR3. While some differences emerged, they could readily be explained by the participants’ varying backgrounds and local contexts. Moreover, the small number of additional topics recommended for inclusion in the list of criteria serves as validation for the questionnaire’s design process. The study also gained from employing a drag-and-drop method for statement ranking, which yielded unambiguous rankings even though it posed a challenge for some participants.

This study also had some limitations, such as the relatively small sample sizes of the four participant groups and its narrow geographic scope, which may impact the generalizability of the findings. As highlighted by certain participants, this study did not encompass aspects of patient selection methods, medication review costs nor its reimbursement, while aspects regarding the MR3 follow-up process may have been evaluated too superficially. Another bias may stem from the differences in professional experience with MRs between the different groups, which could imply that those with more experience have a more practical and realistic view on the subject. Finally, there may have been an apparent bias among those without an interest in MRs because they would have been less inclined to participate in our survey.

Additionally, some of the key elements deemed important to evaluate quality of a MR3 may pose implementation challenges. Statement A1, emphasizing the importance of pharmacist-patient communication, is widely considered crucial, but the implementation of assessing it poses significant challenges. Furthermore a list of key quality evaluation criteria necessitates ongoing maintenance and regular updates to keep pace with evolving knowledge and practices.

### Open questions and future research

The qualitatively ranked statements from this study can function as key elements of MR3 quality standards and can be tested and implemented in diverse settings like self-assessment, peer evaluation, or external audit. Instead of using the full list of criteria for auditing, which can be time-consuming, this study suggests an alternative approach where a random subset of criteria is used, with their frequency weighted according to the rankings from the survey. This approach could improve the efficiency of the auditing process while preventing reactive subversion.

## Conclusion

This study revealed a broad consensus regarding the key elements for assessing the quality of a MR3. There was substantial agreement among the four participant groups about the statements deemed most important within each domain. Eight key statements emerged as essential components that should be included in a comprehensive MR3. These statements encompassed aspects such as 1) using understandable language, 2) explaining the purpose of the review to the patient, 3) addressing the patient’s ability to take medications correctly, 4) discussing the appropriate use of each drug for specific conditions, 5) creating and reviewing a recent and clear medication schedule with the patient, 6) evaluating the ongoing relevance of all drug indications, 7) providing an opportunity for patients to discuss their symptoms with the pharmacist, and 8) considering the patient’s expectations and concerns when developing the treatment plan. Some minor differences were observed, related to the participants’ level of experience. In light of the study’s findings and the additional criteria proposed by the participants, the next step is to develop a quality instrument for medication reviews that is both efficient and effective.

## Data Availability

The raw data supporting the conclusion of this article will be made available by the authors, without undue reservation.
